# Estimating Urban Traffic Patterns through Probabilistic Interconnectivity of Road Network Junctions

**DOI:** 10.1371/journal.pone.0127095

**Published:** 2015-05-26

**Authors:** Ed Manley

**Affiliations:** Centre for Advanced Spatial Analysis (CASA), University College London, Gower Street, London, United Kingdom; University of Warwick, UNITED KINGDOM

## Abstract

The emergence of large, fine-grained mobility datasets offers significant opportunities for the development and application of new methodologies for transportation analysis. In this paper, the link between routing behaviour and traffic patterns in urban areas is examined, introducing a method to derive estimates of traffic patterns from a large collection of fine-grained routing data. Using this dataset, the interconnectivity between road network junctions is extracted in the form of a Markov chain. This representation encodes the probability of the successive usage of adjacent road junctions, encoding routes as flows between decision points rather than flows along road segments. This network of functional interactions is then integrated within a modified Markov chain Monte Carlo (MCMC) framework, adapted for the estimation of urban traffic patterns. As part of this approach, the data-derived links between major junctions influence the movement of directed random walks executed across the network to model origin-destination journeys. The simulation process yields estimates of traffic distribution across the road network. The paper presents an implementation of the modified MCMC approach for London, United Kingdom, building an MCMC model based on a dataset of nearly 700000 minicab routes. Validation of the approach clarifies how each element of the MCMC framework contributes to junction prediction performance, and finds promising results in relation to the estimation of junction choice and minicab traffic distribution. The paper concludes by summarising the potential for the development and extension of this approach to the wider urban modelling domain.

## Introduction

The recent emergence of new, large mobility datasets represents a significant opportunity for transportation researchers and engineers alike. While transportation research has always built heavily on data, never before have transportation datasets been available at the spatial and temporal scale and granularity that they are today. As these datasets move towards tracking individual travellers in near real time, the need for the development of new methodologies for understanding and managing transportation systems becomes even more pressing.

One area where new datasets offer significant promise is in the analysis of routing behaviour. The reduced cost and subsequent proliferation of GPS-equipped (Global Positioning System) devices has enabled the passive collection of large datasets describing the fine-scale routing behaviours of hundreds or thousands travellers, such as those using private vehicles [1, 2], minicabs [3], and bicycles [4, 5]. Yet while route choice analysis is one application of these datasets, other opportunities arise around what these datasets can tell us about the more general nature of traffic patterns.

Traffic patterns are conventionally analysed through the movement of vehicular traffic along a single stretch of road. The arrival of large-scale routing datasets, however, potentially enables a dissection of traffic patterns, allowing us to understand how traffic is moving *between* locations. Large-scale routing datasets allow us to examine links between different parts of the road network, understanding how locations are interconnected and dependent on each other. Using this perspective, traffic along a route can be viewed as consisting of multiple constituent flows, each dependent on a set of past and future locations. This insight offers a number of opportunities for the understanding and estimation of traffic patterns under normal and disrupted conditions.

In this paper, a method is introduced for the estimation of urban traffic patterns from location-to-location interconnectivity. Links between locations are constructed from large-scale routing data, which links together locations based on sequential usage. This interconnectivity of locations on the road network reflects the basis of how traffic flow is generated. The approach introduced in this paper makes use of these relationships, introducing a novel probabilistic model of mobility that simulates urban traffic patterns based on the structure of locational connectivity.

At the heart of this approach is a Markov chain Monte Carlo (MCMC) model, modified for the purpose of traffic pattern prediction. Conventional MCMC is a domain-neutral statistical approach to the modelling and prediction of system behaviour. Predictions are constructed through modelling of probabilistic transitions between across *state space*, a representation of the states of a system. The likelihood of transition between states is assessed through the previously observed relationship between current and next possible states. The potential for a shift between two states is characterised with a *transition probability*, extracted from prior observations. The inclusion of multiple states leads to the construction of a Markov chain, a network link representation of inter-state connectivity. Once the chain is defined, the execution of repeated *random walks* across the Markov chain allow the generation of system-level distributions of system state changes. The distribution generated reflects the nature of the transition probabilities between states within the Markov chain. The MCMC approach has been used across a number of fields, including computational biology, social science and economics.

The use of MCMC approaches within transportation and geographic research goes back some time, being used in a variety of applications. The approach has been used in developing trip matrix distributions [6], analysing migration patterns [7, 8], road safety predictions [9] and in sampling route choice alternatives [10] Of most significance are previous work applying Markov approaches to traffic assignment modelling [11, 12]. These approaches aim to capture the traffic equilibrium state as a Markov chain, enabling the simulation of day-to-day temporal dynamics. However, the application of MCMC has not extended to the incorporation of large-scale mobility datasets for the specification of Markov chains.

This paper introduces a new methodology for the application of MCMC in estimating traffic patterns within urban areas. The work derives transition probabilities for movement between important locations on the urban road network from large granular routing datasets, extracting the inherent interconnectivity between these locations. Across this Markov chain, multiple routes are then generated through the execution of random walks, leading to the production of traffic pattern estimations. In order to achieve the application of MCMC in this context, a number of amendments to the conventional approaches are incorporated. The aim of this paper will be to test whether the novel approach to MCMC is a useful framework for modelling route choice, and whether, in sum, modelled routes are reflective of wider traffic flow.

The paper is laid out as follows. In the next section the modified MCMC approach for predicting urban traffic patterns is introduced, outlining the specification of the Markov chain and amendments to conventional MCMC methods to allow its application in traffic pattern prediction. The third section moves onto the application of the approach, detailing the routing datasets involved and approaches taken in its implementation in London, United Kingdom. Following this, the resulting from traffic modelling is validated against observed movement choice and traffic flow data. In the final section, in addition to summarising the findings, the potential future applications of this approach will be discussed.

## Markov Chain Monte Carlo for Estimating Urban Traffic Patterns

The generic Markov chain Monte Carlo (MCMC) approach describes the relationship between current and future system states. Within the context of estimating urban traffic patterns, aside from specifying the nature of these states and their connectivity, its application towards how traffic estimates are generated requires a number of important further considerations. To achieve this, a number conceptual advances to the conventional MCMC approach are outlined—first, addressing the nature of state space, then addressing how random walks proceed across this space. The implementation of the model outlined here, utilising a large dataset of observed route choices, is described in the next section. Elements of the model framework design outlined in this section will be validated later in this paper.

### State Space and Transition Probability Specification

The state space describes all of the states in which a system, modelled using MCMC, may exist. These states reflect the most important points within the represented systems, from which system transitions may occur. Therefore, in adapting this approach for generating estimates of traffic distributions, the state space should reflect the points in urban space that most strongly influence the traffic redistribution.

In considering urban space, the clearest candidates are major road junctions. These are locations at which senses are heightened and route choices must be considered [13], and offer opportunities for individuals to deviate from their current direction of travel. A route may be characterised as a chain of junctions, with probabilities of connection between each pair, indicative of the dominant flow of traffic around that region. Major junctions will therefore form the state space within the modified Markov chain model.

The calculation of transition probabilities between major junctions is derived from large-scale route choice data. Iterating through each route individually, the major junctions traversed during the route are recorded. Across the entire route dataset, the sum of traversals between each ordered pair of junctions is derived. Transition probabilities are finally calculated according to observed transitions involving other available alternatives, under given prior conditions.

### Incorporation of Prior States

Conventional MCMC models predict transitions between current and future states, minimising the influence of prior context. Within route choice decision-making, however, this bipartite transition may not adequately reflect the momentum inherent in goal-directed travel. Earlier research indicates that there is a human preference for travel along direct and continuous routes [3], suggesting that a prior state may provide an indication of future direction. Within this model, therefore, the prior state (e.g. the previous junction) will be considered along side current states in identifying a future state. The extent to which this element does improve the predictive power of the probabilistic model will be tested during model validation.

The incorporation of momentum is achieved by including the nature of *immediately prior* state (e.g. the previous junction) with the current state in the specification of future state transition probabilities. The implementation of a prior state will capture the sense of directional continuity natural to traffic movement. While further prior states could be included at this stage, these would potentially over fit the model, reducing flexibility outside of predefined paths. The consideration of only current and the immediately prior state retains the indication of momentum while remaining flexible.

**Fig 1:**
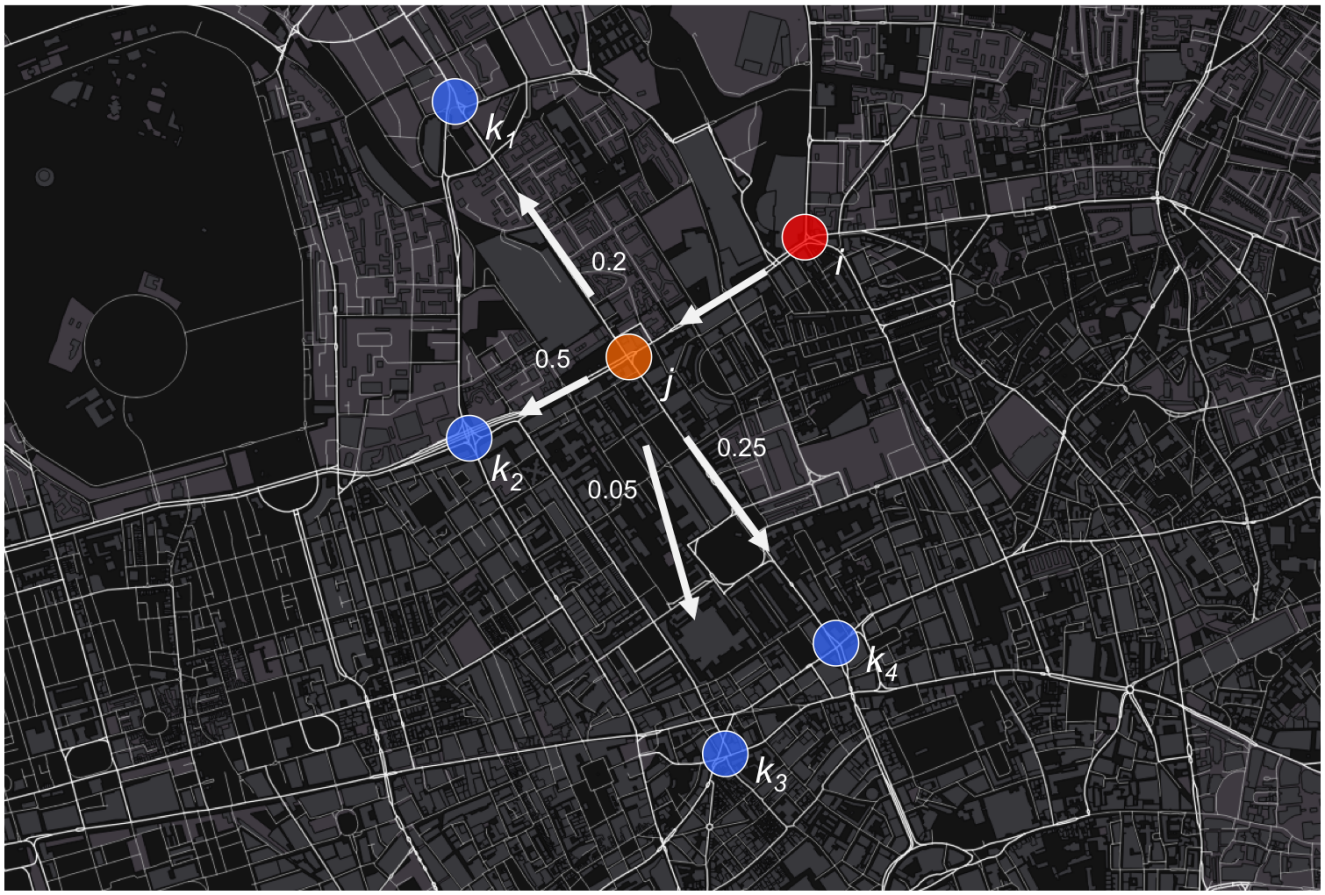
Model of MCMC structure incorporating the prior state (*i*), where major junctions are defined as states and transition probabilities between states given.

A schematic demonstrating the incorporation of the prior state within the Markov chain is outlined in Fig 1. This figure outlines that rather than only considering the current state, *j*, in determining the connectivity between states *j* and all possible future states *k*_*n*_, the prior state, *i*, is implemented too. The probability of transitioning between the current state, *j*, and any of the future possible states *k*_*n*_ is adjusted from *P(i|j)* to *P(k|(j|i)*. In Fig 1, the most likely next state, following previous state *i* and current state *j*, is *k*_*2*_.

This configuration is used as the basis of the construction of random walks across the Markov chain. Given the prior state *i* and the current state *j*, the next state is determined according to a vector of transition probabilities *T* ^*p*^. A state is chosen and the walk progresses iteratively through the state-space until an exit condition is met. In this case, the random walk begins at a junction near to the origin and ends on reaching the junction nearest to the destination.

### Incorporation of Destination Directionality

Within the current configuration of the model, there is a risk that the random walk does not efficiently reach the trip destination. To account for this, the next amendment to the MCMC approach involves the introduction of a weighting towards the direction of the trip destination, used during the generation of random walks. Further to the practical requirements, this amendment also effectively describes elements of the human navigation process, specifically the documented intention to travel broadly in the direction of the trip destination [14, 15, 16]. The weighting amends transition probabilities, adjusting probabilities towards the route destination, and ensuring that the random walk reaches its destination.

In introducing the destination weighting, the vector of transition probabilities (*T* ^*p*^) are multiplied by a vector of deviation probabilities, *T* ^*d*^. This second vector describes the probability of selecting any given junction based on its deviation from the straight-line angle to the destination point. The exact derivation of these values for this case is detailed later. The probability, therefore, of selecting *k* now becomes:

$$P_{k}=\frac{P_{k}^{p}P_{k}^{d}}{\sum_{n}P_{n}^{p}P_{n}^{d}}$$ (1)

Where *k* is a potential future junction, *n* represents all potential future states, *P* ^*p*^ represents the transition probability to that junction, and *P* ^*d*^ represents the probability of converging towards that junction with respect to the direction of the destination.

### Incorporation of Subgoals

A final amendment to the MCMC approach is introduced for the handling of subgoals during random walks. Subgoals in this context are generated for the negotiation of large geographic features over which few points of traversal exist (e.g. rivers and parkland). Given the limited points at which traversal can be made, it is assumed that available crossing points form an initial subgoal via which the routes are directed.

Within the amended MCMC model, a subgoal routine is integrated whereby an initial target destination is set as the crossing point of a geographic feature intersecting origin and destination. The walk will initially navigate to the crossing point, and then, once that sub-goal is complete, onwards towards their destination. Crossing point selections are made probabilistically, based on the origin and destination of the walk, drawn from prior observations within the dataset. This probabilistic process is defined prior to the execution of the random walk across the Markov chain.

## Model Implementation—London, United Kingdom

The MCMC approach is implemented for the metropolitan region of London, United Kingdom. In this section, the datasets and specifications used in constructing the Markov chain and random walk elements of the MCMC model are defined. This implementation represents only one possible configuration of the MCMC model outlined in the last section. This configuration will be tested during model validation in the next section.

### Routing Datasets

The primary requirement for the specification of the Markov chain is a routing dataset describing the interconnections between junctions on the road network. For the London case study this is achieved through a large dataset of minicab GPS traces. Trace data was provided by Addison Lee Limited—the largest private hire minicab company in London—for three months between December 2010 and February 2011. In addition to the GPS data, trip logs were provided enabling identification of the origin and destination of the 690045 trips, undertaken by 2950 drivers, encompassed within the dataset. The Addison Lee business model means origin-destination trips are charged on a fixed rate, meaning minicab drivers are incentivised to minimise their journey times. The routes taken during the course of these journeys will be used to define the Markov chain.

While minicab routes provide only a limited representation of the entire distribution of traffic within London, it is important to note that the main requirement of the dataset is in the specification of the interconnectivity between junction locations. A benefit of the scale of the dataset is that trips are observed across the whole of London. However, a sizeable majority—84%—of the journeys take place solely in inner and central London regions, with 95% of trips having either an origin or destination within inner or central London. While this still leaves over 110000 trips available for the definition of junction connectivity in outer London areas, these definitions will be based on fewer observations and so potentially more open to bias. Further discussion of these limitations will be outlined in later discussions.

In constructing the Markov chain, the dataset is divided into a calibration and validation partitions according to an 80:20 split of randomly sorted routes. The calibration dataset—containing 552036 journeys—will be used for the specification of the Markov chain (described in detail later), with the validation dataset—consisting of 138009 routes—reserved for establishing the accuracy of the final MCMC estimates.

### State Space Specification

State space is defined as the set of major junctions on the London road network. These definitions are drawn from the Ordnance Survey Integrated Transport Network (ITN) dataset, the most comprehensive GIS representation of the UK road network. The extraction of major junctions from this dataset requires two definitions.

The first specification involves the definition of what a major junction encompasses. For London, these definitions follow the road network hierarchy specified by Transport for London and the UK Department of Transport. In this case, only junctions that intersect those roads defined by one of the top two classifications—‘Motorway’ and ‘A-Road’—will be defined as major junctions (US equivalents are ‘Freeways’ and ‘Arterials’; In France, ‘Autoroutes’ and ‘Routes Nationales’ are similar). ‘Motorways’ and ‘A Roads’ carry large volumes of urban traffic and, as such, intersections between these roads can be expected to be salient locations, significant in route decision-making.

The second specification involves the geographic location of these features. A location is required for the angular deviation elements of the model outlined earlier. However, within the ITN dataset, major junctions are often made up of multiple road segments, offering no single location at which the junction exists. To reach a solution, first, all intersecting points between major roads are clustered together where found within a 250-metre radius of each other. The 250-metre buffer ensures that all points at even very large junctions are covered, while differentiating between closely distributed smaller junctions. Once these clusters are established, the centroid of all these points is calculated, defining the single location for that junction. This process yields 816 junction points across the entire London road network.

Assessing observed route flow through this subset of junctions, the strong central London concentration of trips is demonstrated. In the central London areas of Knightsbridge, the South Bank, Soho and the City of London, an average of 4079 trips pass through each junction; outside of these regions the average falls to 365 trips. A maximum trip count of 15980 is observed at the Hyde Park Corner junction between Knightsbridge and Westminster. The increase in trips passing central London is matched, however, with an increase in the spatial density of junctions in these areas.

### Transition Probability Calculation

The specification of interconnectivity between junctions is drawn from the routing patterns described within the minicab route dataset. Routes are analysed with respect to their movement across the junction network, and inter-junction connectivity calculated in terms movements via triplets—prior, current and future—of junctions. These connections are derived from the calibration dataset of 552036 routes, described earlier.

In specifying these probabilities, each path is inspected. Where the individual is observed traversing the network from junction *i* to junction *j* to *k*, the observation augments the probability of movement to all *k*_*n*_ given prior and current junctions *i* and *j*. In other words, in a given situation where movement from junctions *i* to *j* to *k* is observed, the probability of this event occurring in future is increased, and the probability of transitions from *i* to *j* to all other *k*_*n*_ are reduced.

The strength of connectivity between junctions is differentiated by time of day, with transition probabilities calculated separately for journeys taking place during the daytime (7am to 7pm) or evening (7pm to 7am) hours. This distinction aims to capture temporal deviations in movement patterns borne through congestion avoidance at certain times of the day.

Once all inter-junction connections are constructed, a final phase of chain pruning is undertaken. During this phase all connections with a probability of lower than 0.025 are removed from the structure. This is to reduce the potential influence of erroneous connections persisting within the datasets, and increase the overall speed of route computation.

### Destination Deviation

The probability of selecting a junction that deviates from the direction of the destination is again derived from the route dataset. Assessing once more the connectivity between junctions during each journey, the angular deviation that the selection of a given junction represents, relative to the direction of the destination, is calculated.

**Fig 2:**
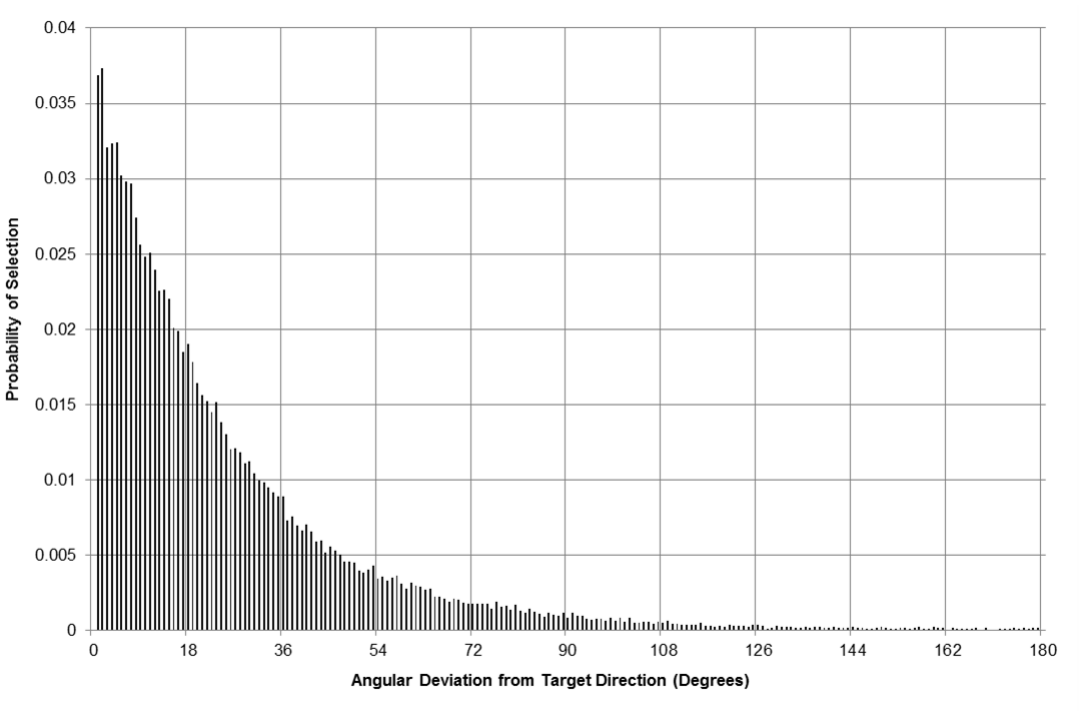
Discrete probability distribution of junction selection based on deviation from destination location.

Running this process for all junction-to-junction selections across the entire dataset, one is able to derive the frequency distribution for junction selection by degree of deviation. A discrete probability distribution is simply derived from this data for each 0.9° shift, generating 200 categories for all absolute deviations up to 180°. This specification does not therefore account for U-turning or backtracking, assuming a continuous direction of travel towards the destination. The derived distribution, shown in Fig 2, would suggest however, that there is a very low probability of choosing a subgoal that deviates more than around 60° from the direction of the destination.

### Subgoal Selection

The final set of estimations is extracted with respect to the traversal of crossing points over waterways in London. London has two main waterways with limited points of traversal—the River Thames (running east to west through central London) and Lea Valley (running north to south in east London). Referring once again to the route dataset, only trips that traverse either of the two rivers are extracted. This subset of cross-river journeys is then aggregated by origin and destination locations, defined at postal region granularity, for extraction of region-to-region bridge choice probability. By breaking down trips origin and destination locations, a link is made between geography of the route and the probability of selecting each possible river crossing.

Examining each journey between an origin-destination pair, the popularity of each bridge, in linking the two regions, is ascertained, and a probability of selecting any specific crossing point derived. These estimates are again calculated separately for both day (7am to 7pm) and evening (7pm to 7am) hours. So, for example, if moving from region W1 to SE16, there exists a 0.35 probability of taking Westminster Bridge, a 0.25 chance of taking London Bridge and a 0.183 chance of selecting Blackfriars Bridge.

### Junction Route Construction through Random Walks

Using the model calibrated to the London road network according to the methods listed above, route simulation can be undertaken through execution of random walks between origin and destination locations. The creation of a route first requires the execution of the MCMC model, creating a junction-based route. The selection of junctions runs in a stepwise fashion, moving from junction-to-junction, with subsequent selections made at each junction. The route continues until the junction nearest to the destination is found. This specific process implemented in achieving this route proceeds as follows:

1. Extract the journey origin and destination point locations on the road network.
2. Identify a start junction from where to begin the journey. Specified as the nearest junction to the origin location falling within a maximum 45° deviation from the straight-line angle between origin and destination locations. The deviation constraint ensures initial choices are directed towards the eventual target.
3. Identify a final junction. Identified as the nearest junction to the destination coordinate.
4. Establish whether start and final junctions are on separate riverbanks. If so, identify a crossing point through probabilistic selection (outlined above). Set the crossing point as an initial subgoal junction on route.
5. Identify the second junction from potential connections from the start junction. Given that a prior junction has yet to be specified, this junction is selected according to its deviation from the straight-line distance between origin and destination junctions. The inverse of the angular deviation from straight line to the destination junction is calculated for each potential junction. The junction with the highest score is set as the next junction, with the start junction then specified as the previous junction.
6. Start iterative junction selection process. At each junction, identify probabilities of successive junctions based on current and prior junction selections (*P* ^*p*^). Calculate angular deviation towards each junction away from straight line towards the destination, and identify corresponding probability of selection (*P* ^*d*^). Using both *P* ^*p*^ and *P* ^*d*^, calculate overall likelihood of selection of each option, according to Eq 1. Assign the probabilities as a weighting for each option, and draw a uniform random number and select option corresponding to the random variable. Move to selected junction, assign current junction as prior. Continue process until final junction is found.

The output from this process is a list of junctions for traversal between origin and destination.

### Road Segment Route Construction

The final stage of the MCMC process involves the construction of a complete route from road segment data. While the route is constructed based on junction-to-junction transitions, traffic data is recorded on a road segment basis. It is assumed that, over the relatively short distances between junctions, route distance can be more effectively minimised than they would over longer distances, as the clarity of the spatial environment becomes less clear. The reduced inter-junction distance furthermore reduces the potential route options that may be available to an individual in traversing the two locations.

In order to test the assumption of junction-to-junction cost minimisation the routing data was again examined. The correlation in routes between short trips, those of less than 1km in distance, and a range of optimal alternatives are established. A 79.8% match is found against equivalent optimal distance routes. High correlation is also observed with other alternatives—including least time (90.40%), lane-weighted time (87.97%) and turn-weighted distance (91.64%) paths—reflecting that over short distances only a limited number of paths are generally available. Given these findings, a shortest distance path assumption is employed in identifying paths between selected junctions.

Road segment routes between junction pairs are generated using a modified version of the A-star network search algorithm. The A-star search approach incorporates a Euclidean distance heuristic into a network search to primarily improve the efficiency of shortest path generation. Rather than searching the entire road network for the optimal route between origin and destination, the minimisation of straight-line distance away from the target location effectively reduces the search space, speeding up path calculation. A small modification is introduced into the method whereby the junction location is specified as the target coordinate, and all roads within a 100-metre radius of that point (indicative of the bounds of the effective view shed around the junction) are specified as potential destination roads. The path search terminates on arrival at a destination road.

### Technical Implementation

The model was written in Java, implementing the JUNG network modelling library and the GeoTools library for spatial association calculations. All junction connection probabilities were pre-processed and queried during route modelling from a locally held MySQL database. Route modelling was executed on a standalone desktop Apple iMac, with a 3.4 GHz Intel Core i7 processor and 16GB RAM.

## Model Results and Validation—London, United Kingdom

The validation stage will examine two dimensions of the MCMC route modelling approach. First, we must assess the ability of the model to predict observed junction-to-junction movement choices, including how each element of the model contributes to the prediction. Second, we must assess how well the complete segment-based routes correspond to the observed set of routes, indicative of traffic flow. Through these stages, the strengths and weaknesses of the approach can be better established.

For validation, junction and route predictions are calculated for the remaining 20% validation partition of the minicab dataset. During this stage, 138009 routes are modelled from observed origin and destination points, observed and predicted junction and route choices are extracted.

### Junction Selection Accuracy

The ability of the MCMC approach to correctly predict the selection of a junction is a highly important indicator of the model’s predictive power. This stage of validation will provide an opportunity to explore the predictive capability of the approach, yet it furthermore allows an exploration of how each constituent element of the model contributes towards prediction.

The accuracy of the junction selection process is judged by assessing how often the model correctly predicts an observed junction selection. For each observed junction choice, information pertinent to that choice (e.g. previous junction, destination directionality, etc.) is extracted and input for the simulation of a corresponding choice using the MCMC approach. Insight is gained by assessing how well the model performs, and the circumstances under which performance varies.

In the first instance, this accuracy test is performed using both the complete model framework and an array of adjusted model configurations. These adjustments to the structure allow an examination of the influence of each element of the model in improving or reducing predictive power. The model adjustments to be tested include *a)* the removal of the prior state assumption (adjusting transition probabilities from *P(k|(j|i)* to *P(i|j)*); *b)* the removal of the destination directionality assumption; *c)* the removal of both the prior state and directionality assumptions; and *d)* the removal of all probabilistic connectivity, accounting for destination directionality alone (junction connectivity is still used in deriving the choice set).

The statistics selected to assess performance describe how well each model performs in the identification and selection the observed chosen junction. In incorporating Precision (Precision = True Positives / (True Positives + False Positives)) and Recall (Recall = True Positives / (True Positives + False Negatives)) measures a better understanding how often the model over predicts or fails to predict a junction is further established (these will become more useful where assessing variation in junction prediction). The performance measures in predicting junction selection are presented in Table 1 for each model configuration.

**Table 1:** Table presenting performance measures for each configuration of the MCMC model.

	Model Configuration				
		A	B	C	D
	Complete	No Previous Junction	No Destination Directionality	No Previous Junction or Destination Directionality	Only Destination Directionality
**Proportion Observed Junction Predicted**	0.58	0.56	0.48	0.27	0.31
**Proportion Observed Junction Most Likely Option**	0.75	0.72	0.67	0.39	0.36
**Mean Number of Alternatives with P>0.1**	2.09	2.15	2.41	3.34	2.80
**Precision Score**	0.58	0.56	0.48	0.27	0.31
**Recall Score**	0.70	0.63	0.47	0.18	0.23

As can be shown in Table 1, performance varies widely across the different arrangements of the modelling framework. Across all metrics, the complete framework demonstrates the strongest performance, closely followed by the model ignoring the role of the previous junction. The complete framework is able to identify the next chosen junction as the most likely on 75% of choices, ultimately being selected at random on 58% of occasions. The results also show that this approach performs best in identifying the chosen option from potential alternatives—producing lower numbers of alternatives with a probability of selection of above 10%, and demonstrating a lower relative impact of false negative selections, indicated by the recall score.

Poor performance is shown by the most ‘stripped down’ approaches. The removal of previous junction momentum and destination directionality assumptions, as in model C, leads to the poorest performance. This indicates that the sense of momentum achieved by including the previous junction is required, and the connectivity of junctions alone is not enough to predict choice. Poor performance is shown again where only considering the direction of the destination, in model D. A better result is achieved where momentum is considered but destination direction is ignored, as seen in model C. These results indicate that inter-junction connectivity contributes more to model performance than destination directionality, but only when both the previous and current junctions are combined in forming the prediction.

In summary, it is clear that probabilistic inter-junction connectivity is a highly important element of the model, but some information around the orientation of the destination is also required. This provides reasonable validation of the proposed framework design. However, doubts remain around the role of incorporating information relating to momentum, through consideration of the previous node. Further tests will explore the performance of the complete model framework in more detail, including where momentum information leads to an improved or reduced model performance.

The second set of junction selection validation tests examines variation in the performance of the complete modelling framework. Specifically, locational variation in performance will be explored, identifying *where* the model performs well, where it does not, and the reasons behind these. These findings may provide insight for subsequent development and improvement of the approach.

For this stage, precision and recall scores are calculated for each junction. These scores indicate where the model is performing accurately, relative to incorrect (false positive, in the case of precision) and missed (false negative, in the case of recall) selections. These results can be mapped to indicate spatial variation across the city.

**Fig 3:**
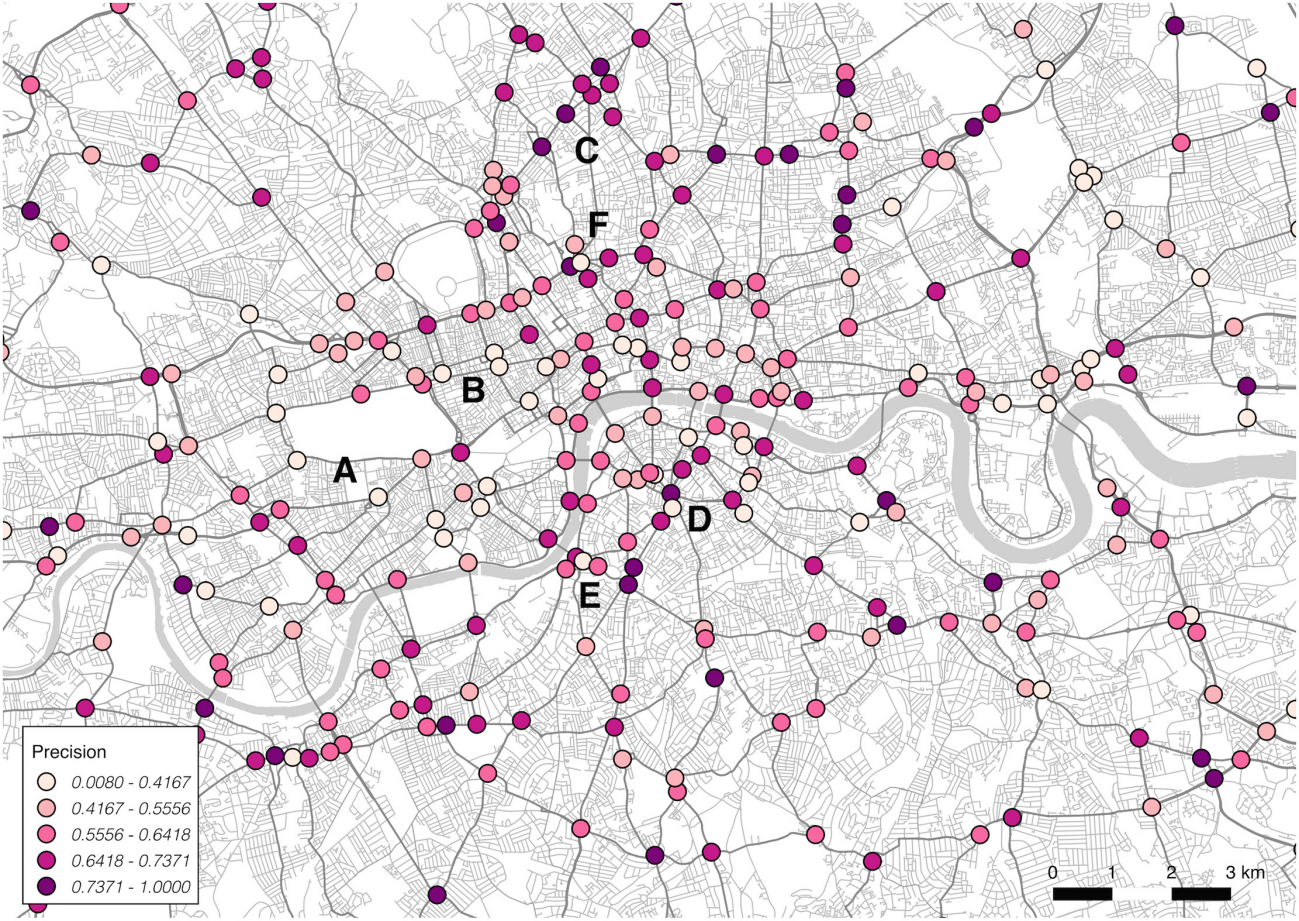
Spatial variation by junction in model precision.

The first map, shown in Fig 3, does demonstrate some spatial variation in precision scores between junctions. This viewpoint highlights positive performance relative to incorrect selection of the junction, achieving a higher score. Precision appears to reduce around the areas marked A and B, in the neighbourhoods of Soho and Knightsbridge. This may be indicative of the nature of spatial environment in granting multiple viable alternative routes. Poor performance is also observed in local cases, marked D, E and F (and observed elsewhere also), where a junction falls close to one or more others. This suggests some interference between junctions that fall near to each other, and may call for a manual simplification of the topology. Strongest performance is observed in the north of the city centre, where precision scores above 0.73 consistency. Again, this may be indicative of the clarity in road network structure.

**Fig 4:**
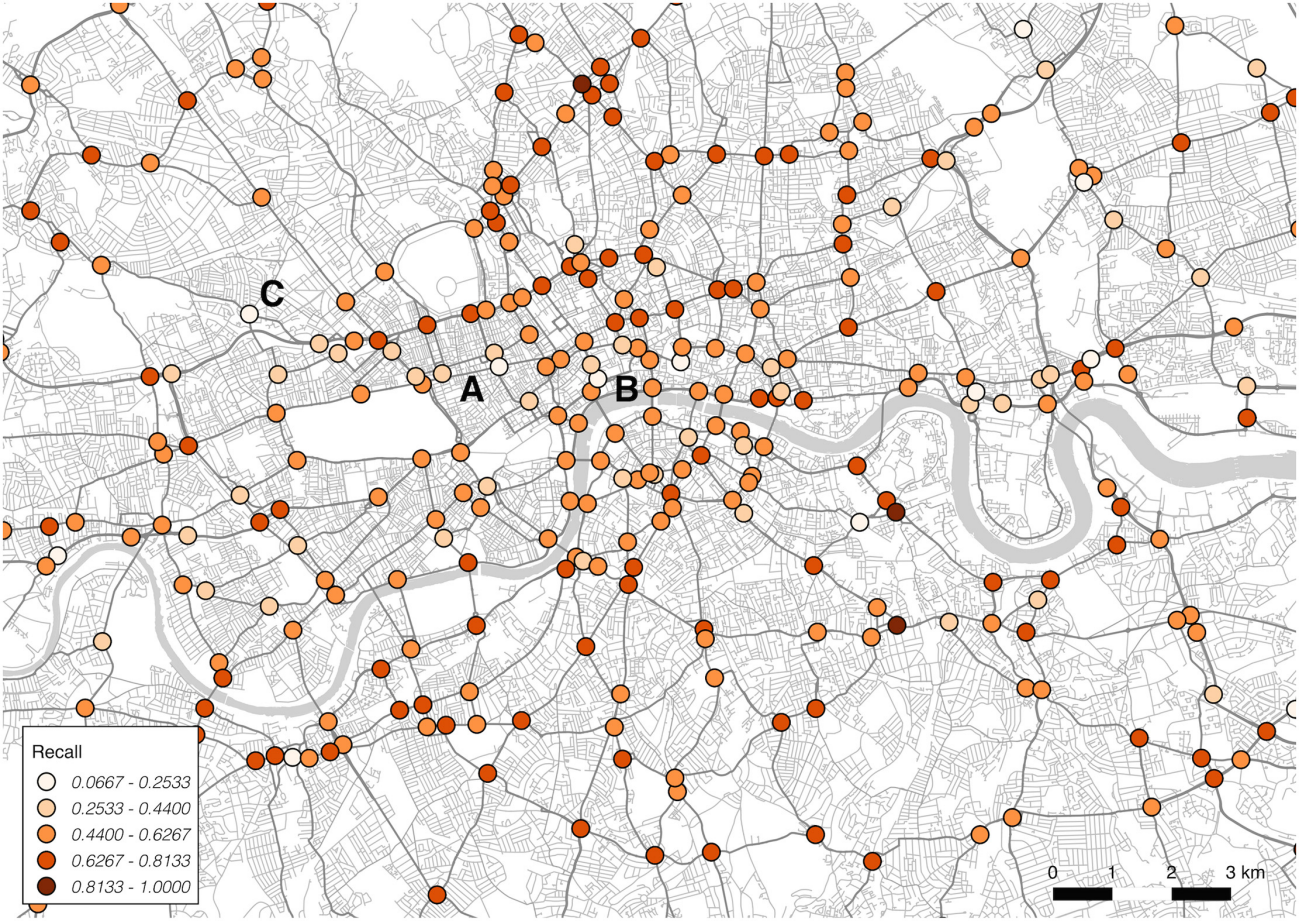
Spatial variation by junction in model recall.

The map showing variation in recall scores, in Fig 4, provides a slightly different viewpoint, measuring junctions that were incorrectly *not* chosen (marked with a lower score). The spread is less strongly spatially correlated, with only a few specific locations—marked A, B and C—showing particularly poor performance. These junctions appear to be ignored in favour of other dominant routes nearby (point C runs close to a dominant thoroughfare running west to east).

**Fig 5:**
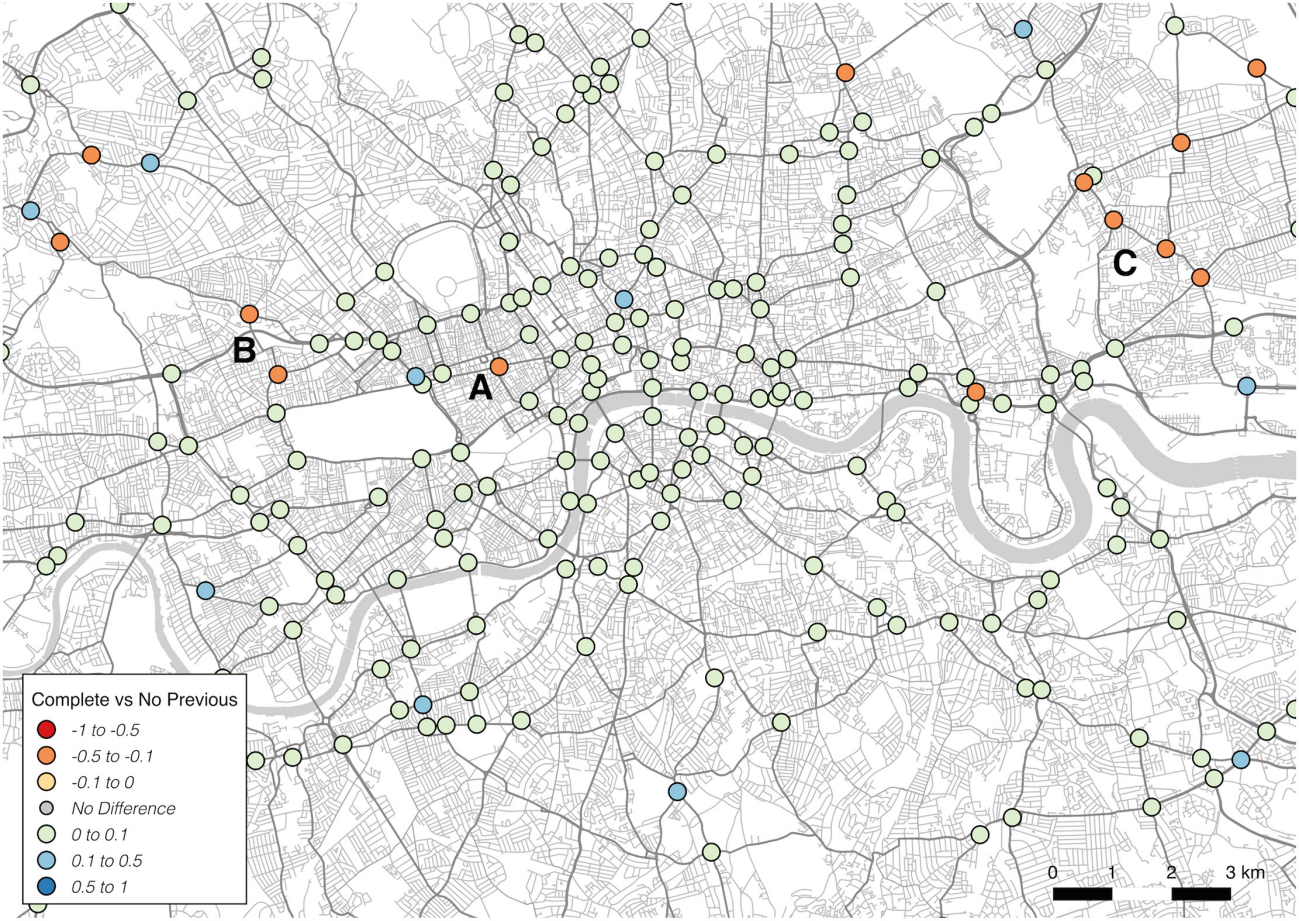
Spatial variation by junction in the difference in precision between complete MCMC model and equivalent model excluding previous junction influence.

Extending the validation of model structure outlined earlier, the third map, shown in Fig 5, highlights how the inclusion or exclusion of the previous junction influences performance. The results indicate how, in the majority of cases, the complete model outperforms the model that does not consider the previous junction. These results suggest that the consideration of momentum in this way is a valid element of the model. However, there are certain cases, marked A, B and C, where momentum appears to cause poor performance. Like the recall results, these again seem to fall close to routes with high traffic flow in a single direction, indicative of the model failing to distinguish local and global patterns of traffic flow. In further work it may be necessary, in accounting for these cases, to investigate how destination directionality can be augmented at certain nodes to better capture deviations from high flowing routes.

### Traffic Distribution Accuracy

The second stage of validation will assess the performance of the complete MCMC approach in predicting the distribution of traffic flow. At this stage, a complete segment-based route is generated for each of the 138009 origin-destination journeys within the validation dataset. The validation will test whether the generated set of routes is adequately reflective of observed patterns of traffic distribution.

It is important to note that observed and modelled routes are not compared against each other during the validation. There are a considerable number of unique behavioural and situational factors involved in real-world route choice, factors that are not captured by this modelling framework. Furthermore, in incorporating prior choices within the model framework, minor deviations in junction choice are likely to be compounded by subsequent selections. It is unfair therefore to argue that deviations between observed and modelled routes are indicative of a poor model fit. Instead, the model should reflect the same general arrangement of traffic, for the given set of origins and destinations, as indicated in the validation dataset.

The MCMC-derived traffic estimations fall across 119360 road segments, with a mean route count of 158.48 trips along each link. Standard deviation in trips along each route is 601.71, with a maximum trip count of 13750, an upper quartile of 38 and a lower quartile of 1. In comparison, the validation dataset shows greater diversity, represented by over 143834 road segments, with a lower mean count of 114.17. The standard deviation of the observed count is 369.65, reaching a maximum at 7684, with the upper quartile at 38 and lower quartile at 1. The Mean Error (ME) of 43.99, indicating a small over-prediction by the model in general.

Examining these errors in absolute terms, the Mean Absolute Error (MAE) arrives at 89.90 with the Root Mean Squared Error (RMSE) considerably higher at 343.27. This difference is indicative of considerable variance in error terms, however, given the similar interquartile range of each dataset, this is likely caused by large variance in traffic on segments with high traffic volumes.

**Fig 6:**
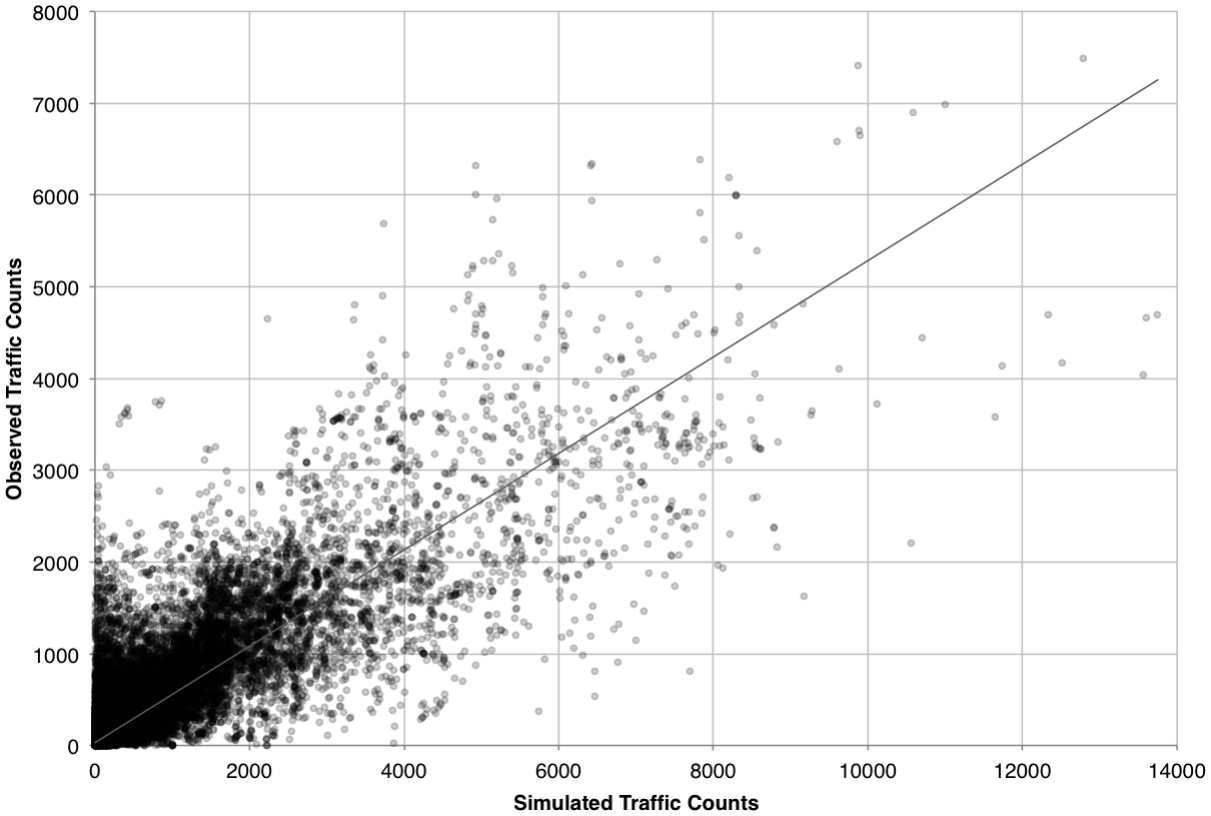
Plot of observed against simulated traffic counts using MCMC approach.

Similarities in route distributions are assessed through plotting a simple linear regression between the two real and modelled datasets, and calculation of the goodness-of-fit between the modelled and real distributions. During this process, the best fitting line was identified between the observed route counts (*y*) and the modelled data (*x*) as falling along the line y = 31.236 + 0.53x. The coefficient of determination (*R* ^*2*^) is found to be 0.74. These indicators would suggest a strong overall prediction of variation within the real data by the modelled representation. The strength of this fit can be observed in the plot between modelled and real values, shown with the line of best fit, in Fig 6.

## Discussion and Conclusions

This paper has introduced a method for the estimation of minicab traffic through the probabilistic interconnectivity of locations on urban road networks. The approach makes use of the increasing availability of large routing datasets that describe fine-scale spatial interactions, building these observations into a Markov chain structure. The paper reported on the implementation of this approach within the London road network, building on minicab route data. This implementation has achieved some promising results. In this section, the significance of these findings, their limitations, and that the potential this approach may hold elsewhere are discussed.

Central to the approach introduced here is the concept that prior behaviours can be used to predict future outcomes. While this is a well-known and well-applied concept within other areas of data-driven research, its application to transportation issues is relatively limited. The model demonstrates how future route choices can be predicted with a reasonably high accuracy based on the major junctions on the road network previously visited. Furthermore, by extending the traditional MCMC approach, improved model performance is achieved by considering both the previous and current junctions visited. The role of destination directionality in shaping route choices was also highlighted. By testing various configurations of the MCMC approach, it was shown how the explicit weighting of inter-junction connectivity by destination direction enhanced performance.

One critique of the approach lies in the assertion that transportation researchers should seek to describe the behaviours being represented within a dataset. This data-driven approach to modelling does not explain behaviour, but captures the inherent heterogeneity and interactions that exist across the road network. While this point certainly has relevance, the increasing availability and range of finely grained mobility datasets will inevitably reduce the need for traditional transport modelling approaches. Exploring how to make complete use of the full granularity within these emerging datasets for transportation research should be a priority. Of course, many large mobility datasets—including that used in the London case study—continue to incorporate a range of biases and limitations. However, as these sources proliferate, the integration of multiple datasets, of varying scale and granularity, will lead towards a more descriptive, more reliable and more comprehensive data representation of the transport system.

Despite positive initial results presented in this paper, a number of methodological elements should be more thoroughly investigated with respect to this specific implementation. One factor to consider is the sensitivity of the destination directionality element. In some cases—such as where using a faster route (such as a motorway) ahead of an alternative—destination direction may be temporarily ignored in order to use a better route. It should be established the locations where destination direction is important, and where is it less so. Similarly, alternative definitions of state space should be given consideration. Road network classifications, although simple to implement, do not necessarily align with all decision-making points or locational salience. Dual network centrality measures or major junctions identified from overall traffic flow offer two potential avenues for exploration.

There are a multitude of directions by which this research may be extended towards the wider urban and transportation modelling domain. In the first instance it would be interesting to explore how well the method performs using a different dataset, particularly one that is more reflective of all traffic flow, rather than only minicab journeys. Another extension may lie in using the routes creating through the MCMC approach in the generation of a choice set between given origins and destinations during discrete choice modelling. Third, the encapsulation of the traffic system as a set of interconnected locations potentially also enables an exploration of network coupling and dependence, within the context of modelling disruption. For instance, in London, according to the minicab dataset, if a traveller has passed two junctions northbound from Kennington Road and along Kennington Lane, there is a 0.92 chance they will continue north onto Elephant and Castle roundabout. This simple insight describes the spatial pattern of dependency upon a location, and provides an indicator of the potential impact were network disruption to occur at that point.

The conceptualisation of the road network as a set of interconnected junctions furthermore potentially helps highlight points of redundancy on the network. Whereas road-level traffic metrics may mask the degree of interactions between two locations, the Markov chain structure of interconnectivity provides greater detail on local level interactions. This potentially allows transport planners to more accurately identify underused routes through the road network, enabling improvements to junction design or the urban realm.

The model presented during this paper has focussed primarily on traffic patterns, yet one clear extension would be its integration with a traffic flow model and trip distribution matrix, enabling the simulation of traffic dynamics. Not only would this enable the simulation of congestion dynamics, but furthermore establish whether the junction interconnectivities derived from the minicab dataset replicate the behaviours of the entire transport network. This simulation framework would additionally allow full scenario testing, such as the closure of a major junction, yielding spatial and temporal estimates of emerging congestion.

While the application of this approach has focussed on road transportation, there are opportunities too for its extension to other transportation domains too. The principle of the interconnectivity of locations extends beyond the road network, and new datasets are becoming available to help capture these patterns. For instance, the distribution of smart cards around urban transit systems may capture probabilistic interactions between origins and destinations. The traces of pedestrian routes may help identify the main locations at which route choices are made, likely reflected some deviations from those used to model vehicular route choice.

Drawbacks in the approach can be associated with its greatest strength, namely its representation of junction interconnectivity. While strongly interconnected junctions reflect high volume interactions between two points, the high strength of connection was shown to override diversity of routing behaviour associated with those junctions. For instance, the probability of turning off a heavily used route to a more minor route was slightly reduced by the continuity of observed distribution along the main road. While the destination directionality measure compensates against this effect to an extent, arriving at a balance between these factors requires further examination.
